# Development of *RAG2* ^-/-^ *IL2Rγ* ^-/Y^ immune deficient FAH-knockout miniature pig

**DOI:** 10.3389/fimmu.2022.950194

**Published:** 2022-08-09

**Authors:** Heng Zhao, Weijian Ye, Jianxiong Guo, Jiaoxiang Wang, Deling Jiao, Kaixiang Xu, Chang Yang, Shuhan Chen, Muhammad Ameen Jamal, Zhongbin Bai, Taiyun Wei, Jie Cai, Tien Dat Nguyen, Yubo Qing, Wenmin Cheng, Baoyu Jia, Honghui Li, Hong-Ye Zhao, Qingfeng Chen, Hong-Jiang Wei

**Affiliations:** ^1^ Yunnan Province Key Laboratory for Porcine Gene Editing and Xenotransplantation, Yunnan Agricultural University, Kunming, China; ^2^ Yunnan Province Xenotransplantation Research Engineering Centre, Yunnan Agricultural University, Kunming, China; ^3^ College of Veterinary Medicine, Yunnan Agricultural University, Kunming, China; ^4^ Institute of Molecular and Cell Biology, Agency for Science, Technology and Research (ASTAR), Singapore, Singapore; ^5^ Department of Physiology, Yong Loo Lin School of Medicine, National University of Singapore, Singapore, Singapore; ^6^ Faculty of Animal Science and Technology, Yunnan Agricultural University, Kunming, China; ^7^ Kunming Institute of Zoology, Chinese Academy of Sciences, Kunming, China

**Keywords:** immunodeficient, pig, liver damage, RAG2, IL2Rγ, FAH

## Abstract

Human hepatocyte transplantation for liver disease treatment have been hampered by the lack of quality human hepatocytes. Pigs with their large body size, longevity and physiological similarities with human are appropriate animal models for the *in vivo* expansion of human hepatocytes. Here we report on the generation of RAG2^-/-^IL2Rγ^-/Y^FAH^-/-^ (RGFKO) pigs *via* CRISPR/Cas9 system and somatic cell nuclear transfer. We showed that thymic and splenic development in RGFKO pigs was impaired. V(D)J recombination processes were also inactivated. Consequently, RGFKO pigs had significantly reduced numbers of porcine T, B and NK cells. Moreover, due to the loss of FAH, porcine hepatocytes continuously undergo apoptosis and consequently suffer hepatic damage. Thus, RGFKO pigs are both immune deficient and constantly suffer liver injury in the absence of NTBC supplementation. These results suggest that RGFKO pigs have the potential to be engrafted with human hepatocytes without immune rejection, thereby allowing for large scale expansion of human hepatocytes.

## Introduction

Orthotopic liver transplantation (OLT) (1) is currently the treatment of choice for patients with end-stage liver disease or liver failure. However, limited availability of donor organs and lifelong need for immunosuppression have limited the number of patients that can benefit from OLT (2, 3). Hepatocyte transplantation (HT) is an alternative to OLT, where donor hepatocytes are engrafted into the recipient’s liver. Compared to OLT, HT is less invasive and cryopreserved hepatocytes can be thawed for use as required. Hepatocytes can also be genetically engineered to correct metabolic diseases or prevent immune rejection, thereby allowing for allogeneic transplantation (1). Nonetheless, clinical adoption of HT is low due to the limited supply of high quality hepatocytes (3). It is estimated that 5-20 billion hepatocytes are required to treat a single case of acute liver failure (4).

To address the availability of hepatocytes for HT, various *in vitro* methods of primary human hepatocytes (PHH) expansion had been developed. Though 2D *in vitro* cultures comprising of supporting nonparenchymal liver cells supported hepatocyte growth, these 2D-cultured hepatocytes are considerably dissimilar to hepatocytes *in vivo* (5, 6). Advances in 3D organoid culture techniques have improved hepatocyte culture allowing for their use in the study of liver diseases, drug metabolism, and gene therapy studies (7). Notably, when grown in 3D organoids, adult PHH can even be expanded (8, 9). Nonetheless, due to the costs and complexity of these systems, it is not feasible to scale-up such technologies to generate the billions of hepatocytes required to repopulate a human liver (5, 10).

An alternative strategy involved the use of immunodeficient mice for *in vivo* expansion of human hepatocytes (11). One of the earliest mouse models to demonstrate a human-mouse chimeric liver is the urokinase-type plasminogen activator (uPA) - recombination activation gene 2 (RAG2) knockout (uPA-Rag2^-/-^) mouse model (12). Continuous hepatic injury from the transgenic expression of uPA driven by the murine albumin promoter, coupled with the absence of mature murine B and T cells, allowed for up 15% of the murine liver to be repopulated by human hepatocytes (12, 13). Further improvement in engraftment efficiency was observed in uPA-Rag2^-/-^Il2rγ^-/Y^ mice, where knockout of the interleukin 2 receptor subunit gamma (IL2Rγ) further impaired the functions of murine natural killer (NK) cells (14). To control the extent of liver injury, fumarylacetoacetate hydrolase (FAH) knockout mice were generated. FAH knockout leads to the toxic accumulation of fumarylacetoacetate in hepatocytes leading to liver damage. However, these mice can be rescued with 2-(2-nitro-4-trifluoromethylbenzoyl)-1,3-cyclohexanedione (NTBC), which blocks tyrosine catabolism upstream of FAH. By cycling NTBC administration, which causes gradual FAH^-/-^ hepatocytes death, the hepatic niche can be opened for rapid repopulation by transplanted human hepatocytes. Utilizing Fah^-/-^Rag2^-/-^Il2rγ^-/Y^ (FRG) mice, it was demonstrated up to 90% human chimerism in murine liver and showed that human hepatocytes can be serially transplanted from a humanized-liver FRG mice to another FRG mouse (15).

Even though high-quality human hepatocytes can be expanded in FRG mice, the small size of mice still limit the scalability of this strategy. In contrast, in terms of genetics, anatomy, physiology, size and lifespan, pigs are closer to humans than small mammals (16, 17). The porcine immune system and its development are also more human-like (18, 19). Therefore, pigs can be excellent animal models for biomedical research, such as the development of humanized tissues and organs for transplantation. Already, pigs that lacked various porcine antigens, such as α-galactose-1,3-galactose, had been genetically engineered to reduce hyperacute xenograft rejection upon human xenotransplantation (20, 21). Pigs that have been genetically engineered to prevent transmission of porcine endogenous retroviruses have also been developed (22, 23). Nonetheless, delayed xenograft rejections and antibody mediated rejections ultimately sets in (21).

An alternative to xenograft transplantation, is the expansion of human cells in immunodeficient pigs. For example, thymectomized and partial hepatectomized mini-pigs had been shown to accommodate transplantation of human hepatocytes for 2-3 weeks (24). Similarly, thymectomized and splenectomized mini-pigs tolerated engraftment of human vascular grafts for up to three months without rejection (25). Though these models demonstrated that surgically produced immunocompromized pigs (SPIP) can potentially support human xenografts, considerable costs and surgical expertise is required, which limit the numbers of SPIP that can be produced. Moreover, rejection by residual functional porcine immune cells prevent achievement of high levels of human chimerism. In contrast, genetically engineered immunodeficient pigs once created, require less expertise in handling, maintenance, and propagation. Various RAG2^-/-^ pigs have been created and they had been shown to tolerate human induced pluripotent stem cells engraftment (26, 27). Similarly, low percentages of human leukocytes can be detected in the peripheral blood and various organs of ART^−/−^ IL2Rγ^−/Y^ SCID pigs engrafted with human CD34^+^ cord blood (28).

Herein, we report on the successful generation of RAG2^-/-^IL2Rγ^-/Y^FAH^-/-^ (RGFKO) pigs through CRISPR/Cas9 editing of fetal fibroblasts followed by somatic cell nuclear transfer (SCNT). The RGFKO pigs had a severely defective immune system companioned with progressive liver damage. The immunodeficient pigs with liver injury will be a good large animal model amenable to human hepatocyte engraftment in future.

## Results

### Generation of RAG2^-/-^IL2Rγ^-/Y^FAH^-/-^ (RGFKO) pigs by somatic cell nuclear transfer

**Figure 1:**
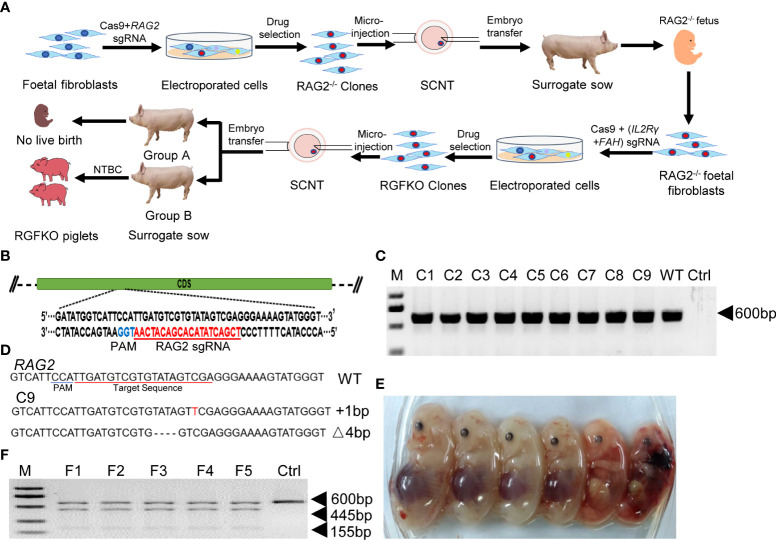
Targeted disruption of RAG2 by CRISPR/Cas9. **(A)** Schematic representation of the workflow to generate RGFKO pigs. RAG2^-/-^IL2Rγ^-/Y^FAH^-/-^ (RGFKO) pigs were generated through sequential mutation of the RAG2 gene to generate RAG2^-/-^ fetus first followed by mutations of IL2Rγ and FAH genes to generate triple knockout RGFKO pigs. To mutate the targeted gene(s), single guide RNA (sgRNA) targeting the respective gene(s) was first designed. Day 33 fetal fibroblasts were then electroporated with sgRNA and Cas9 plasmids, followed by drug selection. Positive clones were used as donor cell for somatic cell nuclear transfer (SCNT). SCNT embryos were then transferred into surrogate sows to establish pregnancy. **(B)** Endogenous RAG2 locus and the sgRNA targeting site are shown. **(C)** Genomic DNA was obtained from nine clones (C1-C9) after puromycin selection. RAG2 was amplified by PCR for further Sanger sequencing. Ctrl: no template control. **(D)** Alignment of Sanger sequencing results of clone C9 with wildtype RAG2 sequence demonstrating a one nucleotide insertion (^+^1bp) and a four nucleotides deletion (Δ4bp) mutation. **(E)** Six fetuses were obtained from surrogate sows transplanted with SCNT embryos using clone C9 as donor nuclei. **(F)** Representative results of T7-endonuclease I assays, and gel shift assays performed on the genomic DNA of fetuses F1- F5. Wildtype genomic DNA was used as control (Ctrl).

RAG2^-/-^IL2Rγ^-/Y^FAH^-/-^ (RGFKO) pigs were generated through sequential mutation of the Rag2 gene to generate RAG2^-/-^fetuses followed by mutations of IL2Rγ and FAH genes (**Figure 1A**). To mutate the RAG2 gene, we first designed a single guide RNA (sgRNA) targeting the coding sequence of RAG2 (**Figure 1B**). RAG2-sgRNA and Cas9 were then co-transfected into day 33 fetal fibroblasts by electroporation. After drug selection, nine single-cell colonies were obtained. PCR products were used to amplify the RAG2 for Sanger sequencing (**Figure 1C**). Genomic sequencing of colony C9 showed that a 1 bp insertion mutation and a 4 bp deletion mutation were detected in RAG2 (RAG2^+^1/-4) (**Figure 1D**). Both mutations were sufficient to cause frame shift mutations in the RAG2 gene, resulting in the production of an inactive RAG2. As such, C9 was used as donor cell for somatic cell nuclear transfer (SCNT). SCNT embryos were then transferred into seven surrogate sows, resulting in six fetuses (F1-F6) (**Table 1**, **Figure 1E**). Fetuses were then harvested for fetal fibroblast isolation. T7 endonuclease I (T7EI) assays performed on the genomic DNA (gDNA) of F1- F5 demonstrated heteroduplexes of mutated DNA (**Figure 1F**). Consequently, F1 fetal fibroblast was used for subsequent mutation of IL2Rγ and FAH genes.

**Table 1:** Summary of the generation of RAG2^-/-^ fetuses by somatic cell nuclear transfer.

Recipients	Donor Cells	Pregnancy^1^ (%)	Duration of pregnancy (d)	No. of fetuses
1	C9 (RAG2^-/-^)	–	–	–
2		–	–	–
3		–	–	–
4		+	35	6^a^
5		–	–	–
6		+	<29	0^b^
7		–	–	–
Total		28.6%		6

^1^: Pregnancy was confirmed using ultrasound scan on day 23.

^a^: fetuses were harvested for fetal fibroblasts.

^b^: Pregnancy was aborted before ultrasound scan on day 29.

**Figure 2:**
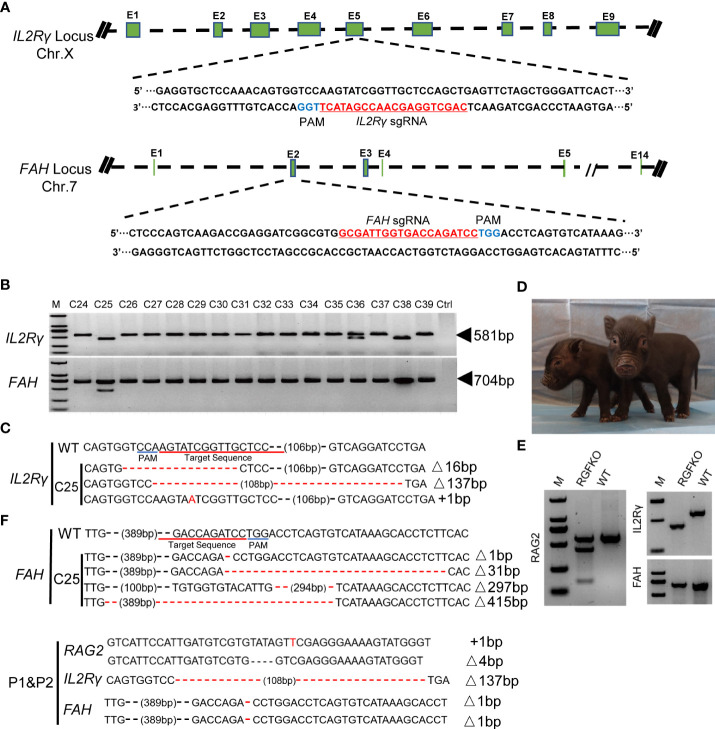
Targeted disruption of IL2Rγ and FAH by CRISPR/Cas9. **(A)** Endogenous IL2Rγ and FAH loci and their sgRNA targeting site are shown. **(B)** Genomic DNA was obtained from 16 clones (C24-C39) after puromycin selection. IL2Rγ and FAH were amplified by PCR for further Sanger sequencing. Ctrl: no template control. **(C)** Alignment of Sanger sequencing results of clone C25 with wildtype IL2Rγ and FAH sequences. 16 and 137 nucleotide deletion (Δ16bp, Δ137bp), and one nucleotide insertion (^+^1bp) mutations were observed in IL2Rγ. Deletion mutations of 1, 31, 297 and 415 nucleotides were observed in FAH (Δ1bp, Δ31bp, Δ297bp, Δ415bp). **(D)** Two live RGFKO piglets (P1 and P2) were obtained after 113 days of pregnancy from surrogate sows transplanted with SCNT embryos using clone C25 as donor nuclei. **(E)** Representative results of T7-endonuclease I assays and gel shift assays performed on the genomic DNA of RGFKO piglet (P1). WT: Wildtype. **(F)** Alignment of Sanger sequencing results of RGFKO piglets (P1 and P2) in the target fragments in RAG2, IL2Rγ, and FAH genes. One nucleotide insertion (^+^1bp) and 4 nucleotide deletional (Δ4bp) mutations were observed in RAG2. 137 nucleotide deletion mutation (Δ137bp) was observed in IL2Rγ. 1 nucleotide deletion mutation (Δ1bp) was observed in FAH.

To mutate IL2Rγ and FAH, Cas9 protein together with sgRNA targeting the fifth exon of IL2Rγ and sgRNA targeting the second exon of FAH (**Figure 2A**), were used to transduce RAG2^-/-^ fetal fibroblasts. After selection, 16 single-cell colonies (C24-C39) were obtained, and the target fragments of IL2Rγ and FAH were amplified by PCR for Sanger sequencing (**Figure 2B**). Colony C25 was shown to carry mutations of both IL2Rγ and FAH genes. Sanger sequencing of C25 revealed that the mutations in the IL2Rγ gene included a 16bp deletion, a 137bp deletion and a 1bp insertion, while those on the FAH gene included deletions of 1bp, 31bp, 297bp and 415bp (**Figure 2C**). All these mutations were verified to result in loss of function frameshift mutations of the respective genes. As such, triply edited colony C25 was used as donor cell for SCNT. SCNT embryos were then transferred into 24 surrogate sows and split into two groups – group A with 12 sows received NTBC while group B with another 12 sows received NTBC supplemented feed (**Figure 1A**). Ultrasonography confirmed pregnancy in seven sows from Group A (pregnancy rate of 58.3%; **Table 2**) and nine sows from Group B (pregnancy rate of 75%; **Table 3**).

**Table 2:** Summary of the generation of RGFKO pigs by somatic cell nuclear transfer in sows without NTBC supplementation (Group A).

Recipients	Donor Cells	NTBC status	Pregnancy^1^(%)	Duration of pregnancy (d)	No. of fetuses	No. of stillborn	No. of live birth
1	RGFKO-C25	Without NTBC	–	–	–	–	–
2			–	–	–	–	–
3			+	<29	0	–	–
4			+	<29	0	–	–
5			+	<29	0	–	–
6			–	–	–	–	–
7			+	32	4^a^	0	0
8			+	29	5^a^	0	0
9			+	34	2^a^	0	0
10			–	–	–	–	–
11			–	–	–	–	–
12			+	32	5^a^	0	0
Total			58.3%		16	0	0

^1^: Pregnancy was confirmed using ultrasound on day 23.

^a^: Fetuses were harvested by surgery.

**Table 3:** Summary of the generation of RGFKO pigs by somatic cell nuclear transfer in sows with NTBC supplementation (Group B).

Recipient	Donor Cells	NTBC status	Pregnancy^1^(%)	Duration of pregnancy (d)	No. of stillborn	No. of mummified fetuses	No. of live birth
1	RGFKO-C25	With NTBC	–	–	–	–	–
2			+	<29	–	–	–
3			+	<29	–	–	–
4			+	113	0	6	2
5			+	99	4	0	0
6			–	–	–	–	–
7			+	<29	–	–	–
8			+	<29	–	–	–
9			+	96	3	0	0
10			+	93-95	11	0	0
11			–	–	–	–	–
12			+	<29	–	–	–
Total			75.0%		18	6	2

^1^: Pregnancy was confirmed using ultrasound on day 23.

Although FAH deficiency in humans and mice were not observed to result in *in utero* fetal death (29–31), knockouts of FAH in pigs have been shown to affect fetal development (32). To verify if FAH knockout similarly affects fetal development in RGFKO pigs, four pregnant sows from Group A were euthanized at days 29-32 of gestation to determine fetal developmental status. All 16 fetuses were observed to have died and showed signs of calcification (**Table 2**; **Supplemental Figure S1A**). Sanger sequencing of PCR products confirmed the presence of the IL2Rγ 137bp deletion mutation and the FAH 1bp deletion mutation (**Supplemental Figures S1B, C**). This showed that loss of function mutation of FAH leads to *in utero* fetal developmental defects in RGFKO pigs.

In contrast to Group A, when pregnant sows were supplemented with NTBC (Group B), fetal development was not arrested at day 32. Out of the seven pregnant surrogate sows, four sows progressed till late gestation. Of these, three sows suffered miscarriages between days 93 to 99 of gestation and 18 stillbirths were produced (**Table 3**; **Supplemental Figure S2**). One sow successfully delivered two live births on day 113 of gestation and 6 mummified fetuses (**Figure 2D**; **Table 3**). gDNA of RGFKO piglets were used to obtain PCR amplicons of RAG2, IL2Rγ and FAH for T7EI assay. As shown in **Figure 2E**, gel shift assay demonstrated heteroduplexes and homoduplexes of mutated DNA. Sanger sequencing further confirmed the presence of the RAG2^+^1/-4 mutations, the IL2Rγ 137bp deletion mutation and the FAH 1bp deletion mutation (**Figure 2F**).

To verify that there were no off-targeting issues in RGFKO pigs, we performed in silico off-target prediction using Cas-OFFinder (33). A total of 4, 2 and 9 off-target sequences (OTS) were predicted for RAG2, IL2Rγ and FAH respectively (**Supplemental Table S2**). Using PCR amplification of the predicted sites and Sanger sequencing, we did not detect off-targeting issues in RGFKO pigs.

These results showed that we have successfully generated triple gene knockout RGFKO piglets.

### RGFKO pigs have defective immune system

RGFKO piglets were supplemented with NTBC for the first three days and raised in standard conditions, which cause significant health stresses to immunodeficient RGFKO piglets. For the first two weeks after birth, milk intake by the piglets were normal. However, from day 29 onwards, growth retardation and systemic weakness in RGFKO piglets were apparent. A RGFKO piglet survived to 29 days and another one survived to 29 days, but the reason of death was unknown.

**Figure 3:**
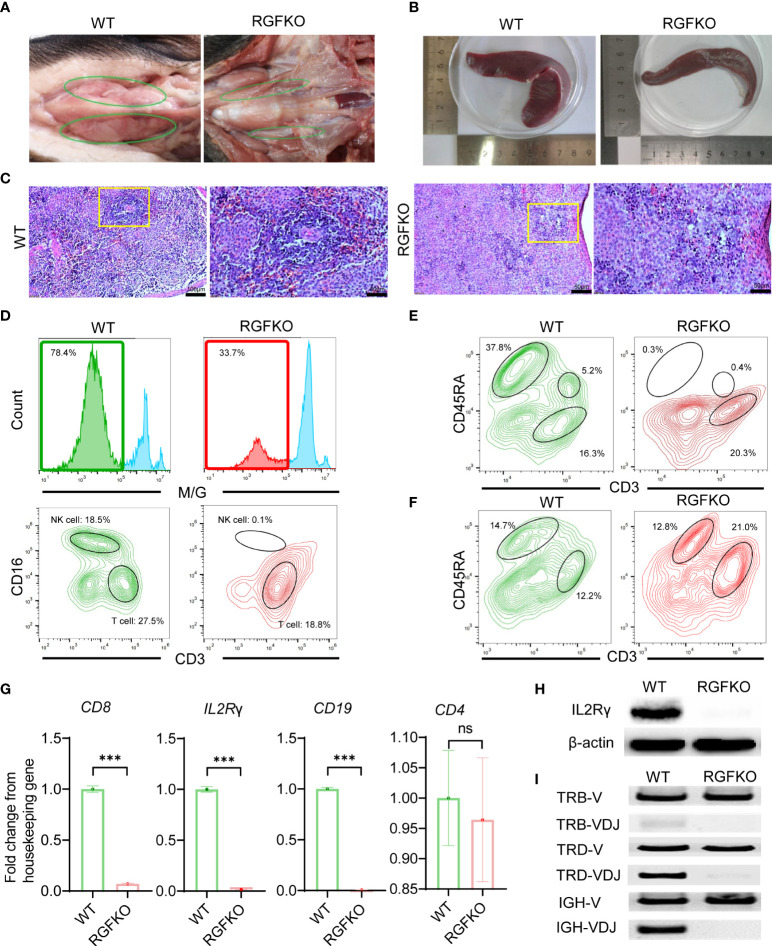
Immunological characterization of RGFKO pigs. **(A)** Representative images of the thymus of wildtype (WT) and RGFKO pigs. **(B)** Representative images of the spleen of WT and RGFKO piglets. **(C)** Representative spleen sections of WT and RGFKO piglets stained with hematoxylin and eosin. Left: normal spleen architecture in WT pig; Right: lacked lymphoid follicles and germinal centers, as well as reduced lymphoid aggregation in the white pulp in RGFKO pig. Boxed region indicating the white pulp region was further magnified. **(D–F)** Flow cytometric analysis of the peripheral blood **(D, E)** and splenocytes **(F)** of WT and RGFKO piglets. **(D)** Shown are representative images of histograms of macrophage/granulocyte (M/G) marker staining. The boxed region, representing M/G negative population, is further gated upon for T cells (M/G-CD3^+^CD16^-^) and NK cells (M/G-CD3-CD16^+^) analysis. **(E, F)** Shown are representative plots of CD45RA against CD3. B cells are identified as CD3^-^CD45RA^+^. Numbers indicate the proportion of the indicated population as a percentage of total peripheral blood mononuclear cells **(D, E)** or splenocytes **(F)**. **(G)** qPCR analysis of the expression of the indicated genes in splenocytes of RGFKO and WT piglets. Shown are fold change of the indicated genes over housekeeping gene (GAPDH). Data shown are mean ± standard error (n = 2 for RGFKO, n = 3 for WT). ns: not significant. ***p < 0.001. **(H)** Western blot analysis of the expression of IL2Rγ in splenocytes of RGFKO and WT piglets. β-actin was used as a loading control. **(I)** PCR analysis of germline TRB-V, TRD-V and IGH-V genes, and V(D)J-recombined TRB-VDJ, TRD-VDJ and IGH-VDJ genes in splenocytes of RGFKO and WT pigs. Shown are representative images of PCR amplification products visualized by DNA gel electrophoresis.

Necropsy of RGFKO piglets and age-matched WT piglets revealed that RGFKO piglets had under-developed thymus compared to age-matched WT piglets (**Figure 3A**). Spleen of RGFKO piglet was also smaller and thinner compared to WT piglets (the weight of RGFKO piglet’s spleen was 4.04 g, while of WT piglet was 5.56 g**;** **Figure 3B**). Haematoxylin and eosin (H&E) staining of the spleen revealed that RGFKO spleen was hypocellular, lacked lymphoid follicles and germinal centers, and had reduced lymphoid aggregation in the white pulp (**Figure 3C**).

To evaluate the changes in lymphocyte populations, peripheral blood mononuclear cells (PBMCs) from RGFKO piglet were harvested. Flow cytometry analysis revealed that the proportion of non-monocyte/granulocyte (M/G) in peripheral blood was reduced from 78% in WT piglet to about 34% in RGFKO piglet. Percentages of CD3^+^CD16^-^ T cells in peripheral blood were reduced from 27.5% in WT to 18.8% in RGFKO piglets. NK cells (M/G^-^CD3^-^CD16^+^) were almost non-detectable in RGFKO piglets (0.1%) compared to WT piglets (18.5%) (**Figure 3D**). CD3^-^/CD45RA^+^ is a well-established marker for porcine mature B cells, which has already been reported by other studies (34, 35). B cells (CD3^-^CD45RA^+^) in the peripheral blood of RGFKO piglets were also dramatically reduced to 0.3% compared to WT (37.8%). As shown in **Figure 3D**, although T cells could be detected in the peripheral blood of RGFKO piglets, no CD3^+^CD45RA^+^ T cells were detectable in RGFKO piglets (**Figure 3E**). This suggested that these T cells were immature, as CD45RA^+^ is highly expressed in mature single positive T cells.

We also investigated the splenic lymphocyte composition. We observed that the percentage of CD3^-^CD45RA^+^ B cells in the spleens of WT and RGFKO piglets were comparable (**Figure 3F**). Given the absence of peripheral B cells, this suggested that a block in B cell maturation in the spleen had occurred. In addition, the percentage of CD3^+^CD45RA^-^ T cells in the spleen of RGFKO piglet were higher (21.0%) than those in WT piglets (12.2%). This most likely represented a population of immature T cells that had escaped thymic clearance and were entrapped in the spleen.

Transcriptional analysis of splenocytes showed that compared to WT, RGFKO piglets have lower expression of CD8 and IL2Rγ mRNA compared to WT. There was no difference in CD4 mRNA expression levels between RGFKO and WT piglets (**Figure 3G**). Western blot analysis using anti-IL2Rγ antibodies performed on splenocytes further demonstrated significant reduction in IL2Rγ expression in RGFKO piglets (**Figure 3H**).

Loss of functional mutation in RAG2 is known to impair V(D)J gene arrangement. As such, we analyzed the degree of V(D)J rearrangements in the T-cell receptor (TCR) and B-cell receptor (34). DNA extracted from spleen were used to detect the rearrangements of loci of TCRβ (TRB), TCRδ (TRD) and immunoglobulin heavy chain (IGH). Although TRB variable (TRB-V) and TRD variable (TRD-V) fragments were detected in both WT and RGFKO pigs but the rearrangement of TRB (TRB-VDJ) and TRD (TRD-VDJ) fragments was reduced in RGFKO pigs (**Figure 3I**). Similarly, the IGH variable fragment (IGH-V) was also detected in both WT and RGFKO pigs but the rearrangement of IGH fragment (IGH-VDJ) was also reduced in RGFKO pigs (**Figure 3I**).

Collectively, the data above showed that RAG2 and IL2Rγ were successfully edited in RGFKO immunodeficient pigs.

### RGFKO pigs have progressive liver damage

**Figure 4:**
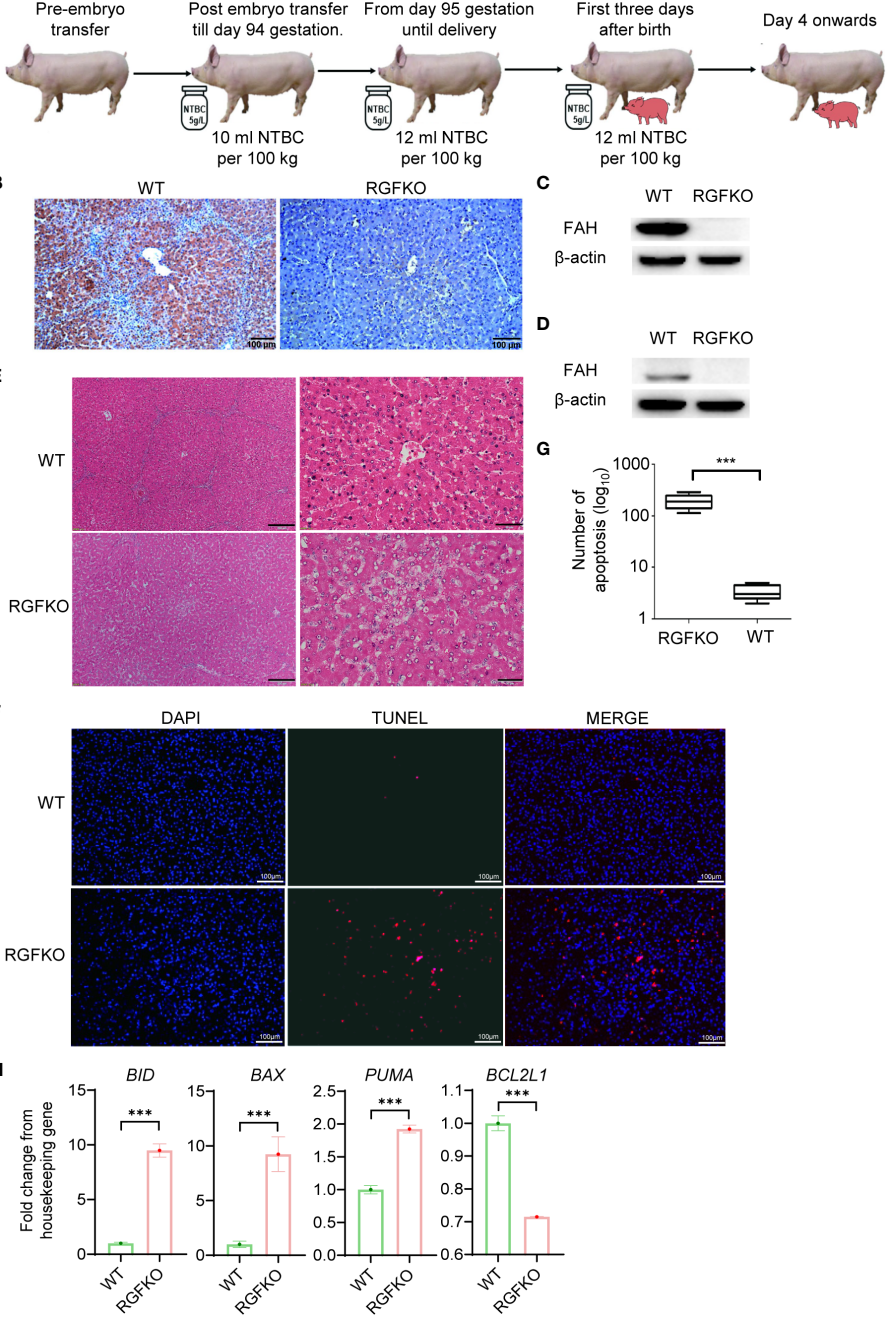
RGFKO pigs suffer hepatic damage when taken off NTBC. **(A)** Schematic of the NTBC dosing regimen for RGFKO pigs. Surrogate sows were supplemented with NTBC (5g/L) at a dose of 10 ml NTBC per 100 kg body weight for the first 94 days of gestation. NBTC dose was increased to 12 ml NTBC per 100 kg body weight thereafter until delivery of RGFKO piglets. RGFKO piglets were supplemented with 12 ml NTBC per 100 kg body weight for the first 3 days after birth. NTBC was then withdrew after that. **(B)** FAH immunohistochemistry analysis of the liver of RGFKO and wildtype (WT) pigs using anti-FAH. **(C, D)** Western blot analysis of the expression of FAH in hepatocytes **(C)** and testes **(D)** of RGFKO and WT piglets. β-actin was used as a loading control. **(E)** Representative liver sections of WT and RGFKO piglets stained with haematoxylin and eosin. Boxed region was magnified to show diffuse hepatocellular injury and cytoplasmic ballooning degeneration. **(F, G)** Evaluation of hepatocyte apoptosis *via* TUNEL assay was performed on liver sections of RGFKO and WT pigs. DAPI (blue) was used to stain the nuclei of hepatocytes, while TUNEL positive cells were stained red **(F)**. TUNEL positive cells were quantified through image analysis **(G)**. **(H)** qPCR analysis of the expression of the indicated genes in hepatocytes of RGFKO and WT piglets. Shown are fold change of the indicated genes over housekeeping genes (GAPDH). Data shown are mean ± standard error (n = 2 for RGFKO, n = 3 for WT). ns, not significant. ***p < 0.001.

Next, we assessed the impact of FAH mutations on RGFKO pigs. To ensure *in utero* development of RGFKO fetuses, surrogate sows were fed feed supplemented with NTBC (5 g/l) at a dose of 10 ml NTBC per 100 kg body weight from embryo transfer up till gestation day 94. Thereafter, NTBC dose was increased to 12 ml NTBC per 100 kg body weight. RGFKO piglets were also supplemented with NTBC at a dose of 12 ml NTBC per 100 kg body weight for the first three days after birth (**Figure 4A**).

Morphologically, the livers of RGFKO pigs did not show obvious pathologies such as tumors or nodules. Next, we analyzed the histology of livers from RGFKO pigs. We first confirmed the absence of FAH in RGFKO livers by immunohistochemistry (IHC) (**Figure 4B**). Western blot analysis using anti-FAH antibodies performed on liver homogenates demonstrated loss in FAH expression, thereby corroborating with the IHC observations (**Figure 4C**). Similarly, loss in FAH expression in the testes of RGFKO piglets was also observed (**Figure 4D**). H&E staining of RGFKO livers showed diffuse hepatocellular injury and cytoplasmic ballooning degeneration. Liver architecture was also disrupted with no clear demarcation of liver lobules (**Figure 4E**). TUNEL assay performed on RGFKO liver sections indicated diffuse staining of numerous apoptotic cells, which were absent in wildtype liver (**Figures 4F, G**). Transcriptional analysis of hepatocytes demonstrated upregulation of apoptotic genes, such as BID and downregulation of BCL2L1, an anti-apoptotic gene (**Figure 4H**). Liver function analysis of pig blood showed that alanine aminotransferase (ALT), aspartate aminotransferase (AST), total bilirubin (TBIL), direct bilirubin (DBIL) in RGFKO piglet were higher than those in WT piglets, while the alkaline phosphatase (ALP), total protein (TP) and albumin (ALB) were lower than WT (**Table S6**). In sum, the above data showed that the loss of function mutation in FAH resulted in the apoptosis of hepatocytes, which subsequently led to hepatocellular damage.

## Discussion

In this report, we successfully generated RAG2^-/-^IL2Rγ^-/Y^FAH^-/-^ (RGFKO) triple knockout *Diannan* miniature pig by SCNT through a two-step sequential mutation of RAG2 followed by IL2Rγ and FAH by CRISPR/Cas9. Consistent with previous reports of RAG2^-/-^ (27, 36, 37), IL2Rγ^-/Y^ (35, 37), RAG2^-/-^Il2rγ^-/Y^ in Landrace cross large white pigs (38), and RAG1^-/-^ IL2Rγ^-/Y^ FAH^-/-^ in Landrace cross large white pigs (39), we observed that RGFKO pigs had hypotrophied or absent thymus, hypocellular spleen, reduced numbers of T, B and NK cells, as well as defective V(D)J recombination. In contrast to single RAG2^-/-^ or IL2Rγ^-/Y^ knockout pigs where no differences in spleen size was observed compared to wildtype (36, 37, 40), the spleens of RGFKO pigs were notably slimmer and smaller. Interestingly, we observed that at a transcriptional level, in contrast to CD8, CD19 and IL2Rγ whose expression were reduced, CD4 expression was not significantly different between RGFKO and wildtype pigs. This is consistent with the observation in RAG2^-/-^ mice, where CD4 expression was not significantly different from wildtype mouse (41). Similarly, CD4 mRNA expression was also not reduced in RAG2^-/-^ pigs compared to wildtype pigs (26).

In B cell development, CD45RA is expressed from pro-B stages onwards (42). VDJ recombination occurs at the pre-B stage, and successful VDJ recombination results in the progression of pre-B into immature B-cell. Immature B-cell then travel to the spleen where they undergo further maturation before entering the peripheral blood. We observed the presence of CD45RA^+^ B cells in the spleen but not in the periphery. This suggested that there is a block in B cell development from the pre-B stage onwards, which is consistent with the loss of VDJ rearrangement (43). Consequently, B cell maturation in the spleen is impaired, resulting in their sequestration in the spleen. In contrast to B cells, most thymocyte T cell populations are CD45RA^-^. VDJ recombination occurs in DN3 stage and is required for thymocyte maturation into single positive T cells. CD45RA expression is only increased at the final step of T cell maturation in the thymus (44). We observed that T cell populations in both peripheral blood and spleen were CD45RA^-^. This supports the notion that T cell maturation has been arrested in the immature thymocyte stages. These immunological changes along with the reduced rearrangements of VDJ TCR or IGH fragments strongly suggest the RAG2 inactivation. Coupled with the absence of NK cells and the significant reduction in IL2Rγ expression at both transcript and protein levels indicate the successful knockout of RAG2 and IL2Rγ.

In addition to the defects in the immune system, FAH expression in RGFKO pigs is dramatically reduced. We showed that consistent with the observations of (32), absence of NTBC supplementation during gestation led to *in utero* fetal death by day 30 of pregnancy, whereas gestational NTBC supplementation rescued RGFKO fetuses. When NTBC is withdrawn after birth, RGFKO suffered liver damage characterized by cytoplasmic ballooning degeneration and disruption of liver architecture.

However, by combining immunodeficiency with liver injury, the challenges involved in maintaining RGFKO pigs are also doubled. Firstly, the RAG2^-/-^ genotype is associated with a high rate of still-births when housed in conventional living conditions (27, 36). Secondly, due to the absence of FAH, NTBC administration during gestation needs to be appropriately titrated to support *in utero* fetus development (32). As such, out of 26 RGFKO fetuses, only two live RGFKO piglets were successfully delivered. When housed under conventional living conditions, the lifespan of RAG2^-/-^immunodeficient pigs were reported to be between 1-3 months (27, 36, 37). Here, we report that RGFKO pigs survived up till one month under conventional housing conditions. The shorter lifespan of RGFKO pigs was probably exacerbated by NTBC withdrawal after the third day of birth. As reported earlier that taking off NTBC FAH^-/-^ succumbed of pigs within 20 days (32). With gnotobiotic living conditions (38) and continual supplementation of NTBC (32), survival of RGFKO pigs is expected to be vastly improved. Nonetheless, an important aspect of this study is that we have demonstrated the feasibility of producing an immunodeficient liver-damage pig model, which represents the foundational steps towards the future development of a humanized liver in pigs.

RGFKO pigs have the potential to be an important source of high quality human hepatocytes for hepatocyte transplant as well as *ex vivo* hepatocyte-directed gene therapy (45). *Ex vivo* hepatocyte-directed gene therapy to correct inborn errors of metabolism had been clinically evaluated in patients with familial hypercholesterolemia with modest results (46). This was mainly due to the poor engraftment rates from low-quality hepatocytes after *in vitro* culture and selection (46). To overcome the need for *in vitro* culture, transduction autologous hepatocytes from FAH^-/-^ pigs with lentivirus expressing FAH, and directly transplanted them back into their autologous host without further culture. They showed that these pigs can be taken off NTBC and thrive with minimal evidence of tumorigenicity and liver damage (47, 48). With the successful generation of our RGFKO pigs, human hepatocytes can now be engrafted into the livers of RGFKO pigs. This opens the possibility of using RGFKO pigs as a large animal model for evaluation of human hepatocyte-directed gene therapy, as well as evaluation of the efficacy of hepatocyte transplants using PHH and liver stem cells.

We have previously improved the safety of porcine xenografts through the inactivation of porcine endogenous retroviruses (22). To reduce the risk of human rejection of porcine xenograft, we have also developed pigs with knockout of porcine α-1,3-galactosyltransferase (20). By allowing the humanization of the porcine liver with human hepatocytes, RGFKO pigs can potentially reduce the amount of remaining porcine tissue in the porcine liver. Given the similarities in porcine and human liver, in terms of size, anatomy and vascular architecture, RGFKO pigs can not only serve as a source of human hepatocytes but also represent the next step forward towards pig-to-human liver transplantation.

In addition to human hepatocyte engraftment, FRG mice have also been used to study immune modulation of human liver disease *via* engraftment with human hematopoietic stem cells (HSC) (49). Such dual liver and immune humanized mice have contributed to the understanding of how the immune system mediate liver inflammation and fibrosis in non-alcoholic fatty liver disease (50) and hepatitis B virus infection (51, 52). In addition to the potential use of RGFKO pigs as human hepatocyte “cell factory” (45), RGFKO pigs can also be engrafted with human HSCs to generate dual humanized immune and liver pigs. Indeed, earlier works using RAG2^-/-^ or IL2Rγ^-/Y^ pigs have shown that immunodeficient pigs are amenable to human cell engraftment (27, 28). Similarities between human and porcine immune-related genes (53), as well as hematopoietic cytokines and immune signaling molecules (54), suggests that human immune lineages might differentiate better in pigs compared to mice. Given the role of the human immune system in various hepatic pathologies, such as hepatocarcinoma (55, 56), non-alcoholic steatohepatitis (50), and hepatitis (51, 52), such dual humanized pigs can thus see applications as preclinical models for evaluation of human-specific therapies, such as chimeric antigen receptor cell therapy and immune checkpoint therapy.

In conclusion, we successfully produced the RGFKO pigs by targeted disruption of the RAG2, IL2Rγ and FAH gene. The RGFKO pigs had a severely defective immune system companioned with progressive liver damage. These pigs will be value for establishing the platform of liver transplantation, stem cell therapies and immunotherapies.

## Materials and methods

### Experimental animals

Animals used in this study were *Diannan* miniature pigs. All animal experiments were approved by the Animal Care and Use Committee of Yunnan Agricultural University in China.

### Chemicals

All chemicals used were obtained from Sigma Chemical Corp (Saint Louis, MO, USA).

### *In vitro* maturation of oocytes

Porcine ovaries were collected from Hongteng abattoir (Chenggong Ruide Food Co., Ltd, Kunming, Yunnan Province, China), and cumulus-oocyte complexes (COCs) with at least three layers of compacted cumulus cells were collected from ovarian follicles of 3-6mm diameter. 50 oocytes were cultured in 200 µL microdrops of TCM199 medium supplemented with 0.1 mg/mL pyruvic acid, 0.1 mg/mL L-cysteine hydrochloride monohydrate, 10 ng/mL epidermal growth factor, 10% (v/v) porcine follicular fluid, 75 mg/mL potassium penicillin G, 50 mg/mL streptomycin sulfate, and 10 IU/mL equine chorionic gonadotropin (eCG) and human chorionic gonadotropin (hCG; Teikoku Zouki Co., Tokyo, Japan) and incubated at 38.5 °C with 5% CO_2_ in 100% humidity for 42-44 h.

### Somatic cell nuclear transfer and embryo transfer

SCNT was performed as previously described (57). After culturing for 42-44 h, oocytes with expanded cumulus cells were briefly treated with 0.1% (w/v) hyaluronidase and enucleated by gentle aspiration of the first polar body and adjacent cytoplasm using a bevelled pipette in Tyrode’s lactate medium supplemented with 10 µM HEPES, 0.3% (w/v) polyvinylpyrrolidone, 10% FBS, 0.1 µg/mL demecolcine and 5 µg/mL cytochalasin B. A single RAG2^-/-^ or RGFKO donor cell was inserted into the perivitelline space of an enucleated oocyte. Donor cell was fused with the recipient cytoplasts with a single direct current pulse of 200 V/mm for 20 µs using an embryonic cell fusion system (ET3, Fujihira Industry Co. Ltd., Tokyo, Japan) in fusion media [0.25 M D-sorbic alcohol, 0.05 mM, Mg(C_2_H_3_O_2_)_2_, 20 mg/mL BSA, and 0.5 mM HEPES (free acid)]. The reconstructed embryos were cultured for 2 h in PZM-3, activated with a single pulse of 150 V/mm for 100 ms, and then cultured in PZM-3 supplemented with 5 µg/mL cytochalasin B for 2 h at 38.5 °C with 5% CO_2_, 5% O_2_ and 90% N_2_. Thereafter, reconstructed embryos were maintained in PZM-3 under similar conditions. Reconstructed embryos cultured for 6-30 h after activation were surgically transferred to the oviducts of the estrous surrogate mother. Pregnancy was first confirmed at approximately 21-29 days after transfer using an ultrasound scanner.

### Design of sgRNA

sgRNAs targeting the coding sequence of RAG2, fifth exon of IL2Rγ, or second exon of FAH, were designed using CRISPOR (http://crispor.tefor.net/) (**Table S1**) (58). sgRNA sequences were then cloned into pGL3-U6-sgRNA plasmids (Addgene no: 51133).

### Transfection of porcine fetal fibroblast

Isolation of porcine fetal fibroblasts (PFFs) were as performed as previously. To generate RAG2^-/-^ PFF and RGFKO PFF, 10 µg of pST1374-NLS-flag-linker-Cas9 plasmids (Addgene no: 44758) and 5 µg pGL3-U6-RAG2-sgRNA plasmids, and 10 µg of pST1374-NLS-flag-linker-Cas9 plasmids, 5 µg pGL3-U6-IL2Rγ-sgRNA and 5 µg pGL3-U6-FAH-sgRNA plasmids respectively were used to transfect 3 x 10^5^ PFF using 4D-Nucleofector (Lonza) as per manufacturer’s protocol. Transfected PFFs were recovered in DMEM supplemented with 10% FBS and incubated at 38 °C with 5% CO_2_. After 48 h, 2 µg/ml of puromycin were added to the medium for 24−48 h to select successfully transfected cells. The survived cells were digested and about 100 cells were seeded into 100-mm-diameter culture dish for 8 days. After about 9 days, puromycin-resistant colonies were picked, and single-cell colonies were transferred to 96-well plates for expansion. When cell confluence reached 70-80%, a portion of the cells were harvested for PCR analysis.

### Genomic sequence validation

DNA were extracted from cells, fetuses or ear tissues of piglets using TIANamp Genomic DNA Kit (TIANGEN, China, DP304). Touchdown PCR was used to amplify RAG2, IL2Rγ and FAH using primers shown in **Table S3**. A portion of PCR amplicons were used for T7 endonuclease I assay (Vazyme, China). Remaining PCR amplicons were cloned into pMD19T (Takara, Japan) *via* TA-cloning and sent for Sanger sequencing. DNA sequences were analyzed using SnapGene software (GSL Biotech, version number: v3.2.1.0).

### CRISPR/Cas9 off-target analysis

Potential off-target sites were predicted using Cas-OFFinder (http://www.rgenome.net/cas-offinder/) (**Table S2**) (33). PCR was used to amplify the predicted regions using DNA obtained from RGFKO ear tissues. PCR amplicons were sent for Sanger sequencing to determine if there were off-target issues.

### Histology, immunohistochemical staining and tunel assay

Liver and spleens of RGFKO pigs and age-matched wildtypes were harvested, fixed in 4% paraformaldehyde for 48 h, and embedded in paraffin. 3-5 mm thick sections were prepared for hematoxylin and eosin and immunohistochemical staining. Anti-FAH antibodies (ABClonal, China) were used to stain for FAH in liver sections at 1:100 dilutions. Images were acquired using BX53 biological microscope (Olympus, Japan). Tunel assay was performed using Tunel BrightRed Apoptosis Detection Kit (Vazyme, China, A113-03) as per manufacturer’s protocol.

### Flow cytometry

Peripheral blood mononuclear cells (PBMCs) were obtained from RGFKO pigs and stained with CD45RA-PE (Abd Serotec, USA, MCA1751PE), CD16-AF647 (Abd Serotec, USA, MCA1971A647), CD3-FITC (Abd Serotec, USA, MCA5951F) and Monocyte/Granulocyte panel-PE (ThermoFisher, USA, MA5-28824) in FACS buffer (PBS supplemented with 0.5% BSA). Data acquisition and analysis was performed using Beckman CytoFlex flow cytometer (Beckman Coulter, USA). Isotype-matched control antibodies were used for all fluorochrome-isotype combinations.

### Reverse transcriptase-quantitative PCR (RT-qPCR)

For quantification of mRNA expression by RT-qPCR, RNA from splenocytes were extracted using TransZol Up (TransGen Biotech, China, ET111-01) according to manufacturer protocol. RNA was then reverse transcribed using PrimeScript™ RT reagent kit with gDNA Eraser (Takara, Japan, RR047A) according to manufacturer protocol. cDNA was diluted ten times and used for qPCR using TB Green Premix Ex Taq II (Takara, Japan, RR820). Primers against IL2Rγ, CD4, CD8, CD19 and GAPDH were used by (59) (**Table S4**). Primers against BID, BAX, PUMA and BCL2L1 were used by (27). The transcript abundance of the various markers was normalized to that of the housekeeping gene, GAPDH using the relative quantification method. The qPCR reaction was performed on the CFX96 Thermal Cycler (Bio-Rad, USA).

### Western blot and protein visualization

Hepatocytes were homogenized in RIPA lysis buffer RIPA lysis buffer (Bestbio, China), separated by SDS-PAGE and transferred to a polyvinylidene difluoride membrane by wet transfer. The proteins were blotted with primary antibodies against FAH (ABclonal, China, A13492, 1:1000 dilution), IL2Rγ (ABclonal, China, A1829, 1:1000 dilution) and β-actin (Zen BioScience, China, 200068-8F10, dilution 1:5000) and further probed with secondary antibodies conjugated with horseradish peroxide enzyme. The proteins were visualized with EasySee^®^ Western Blot Kit (TransGen Biotech, China) on a ChemiDoc MP (Bio-Rad, USA).

### Detection of V(D)J rearrangement

PCR amplifications of TRB-V, TRB-VDJ, TRD-V, TRD-VDJ, IGH-V and IGH-VDJ was performed on splenocyte DNA. The already described primers (37) were validated and used **(** **Table S5** **)**.

### Detection of liver function

The whole blood of RGFKO and WT pigs was collected and the liver function was detected in the first people’s Hospital of Kunming, Yunnan Province (**Table S6**).

### Statistical analysis

All statistical analysis was performed using Graphpad Prism 8.0.2. Unless otherwise stated, numerical data is presented as mean ± standard error of mean (SEM). For single comparison between two groups unpaired t-test was used.

## Data availability statement

The datasets presented in this study can be found in online repositories. The names of the repository/repositories and accession number(s) can be found in the article/**Supplementary Material**.

## Ethics statement

The animal study was reviewed and approved by Animal Care and Use Committee of Yunnan Agricultural University in China.

## Author contributions

Conceptualization, H-JW, QC, and H-YZ. Methodology, HZ, H-JW, and JG. Performance of experiments, HZ and JG. Results interpretation, HZ, WY, QC, and H-YZ. Writing, HZ, WY, KX and MAJ. Resources, H-JW and QC. All authors contributed to the article and approved the submitted version.

## Funding

This work was supported by National Key R&D Program of China (Grant No. 2019YFA0110700), Major Science and Technology Project Yunnan Province (Grant No. 202102AA310047) and by the National Research Foundation Singapore Fellowship (NRF-NRFF2017-03) to QC.

## Conflict of interest

The authors declare that the research was conducted in the absence of any commercial or financial relationships that could be construed as a potential conflict of interest.

## Publisher’s note

All claims expressed in this article are solely those of the authors and do not necessarily represent those of their affiliated organizations, or those of the publisher, the editors and the reviewers. Any product that may be evaluated in this article, or claim that may be made by its manufacturer, is not guaranteed or endorsed by the publisher.

## Supplementary material

Supplementary Figure 1 FAH-deficiency leads to *in utero* fetal death without NTBC supplementation. Without NTBC supplementation during gestation, no RGFKO piglets were born. **(A)** Representative images of fetuses retrieved from pregnant sows implanted with RGFKO embryos at 29-35 days of gestation without NTBC supplementation. **(B, C)** Genomic DNA was obtained from 12 fetuses (F1-F12). RAG2, IL2Rγ and FAH were amplified by PCR **(B)** and amplicons of IL2Rγ and FAH were sent for Sanger sequencing. **(C)** Alignment of Sanger sequencing results of F3, F5, F8 and F12 with wildtype IL2Rγ and FAH sequences. 137 nucleotide deletion mutation (Δ137bp) was observed in IL2Rγ, while 1 nucleotide deletion mutation (Δ1bp) was observed in FAH.

Supplementary Figure 2 Stillbirths of RGFKO piglets. Images of stillbirths of RGFKO piglets from three surrogate sows implanted with RGFKO embryos at 93-99 days of gestation with NTBC supplementation.

Supplementary Table 1 sgRNA target sequences for RAG2, IL2Rγ and FAH.

Supplementary Table 2 Off-target sequences (OTS) predicted for RAG2, IL2Rγ and FAH.

Supplementary Table 3 Primer sequences used to amplify RAG2, IL2Rγ and FAH.

Supplementary Table 4 Primer sequences used for qPCR.

Supplementary Table 5 Primer sequences used for V(D)J rearrangement detection.

Supplementary Table 6 Biochemical function of liver.
