# Supplementary figures and images for: Development of RAG2 -/- IL2Rγ -/Y immune deficient FAH-knockout miniature pig
Source: Front Immunol. 2022 Aug 9;13:950194. doi: 10.3389/fimmu.2022.950194 (PMC9400017; doi:10.3389/fimmu.2022.950194)

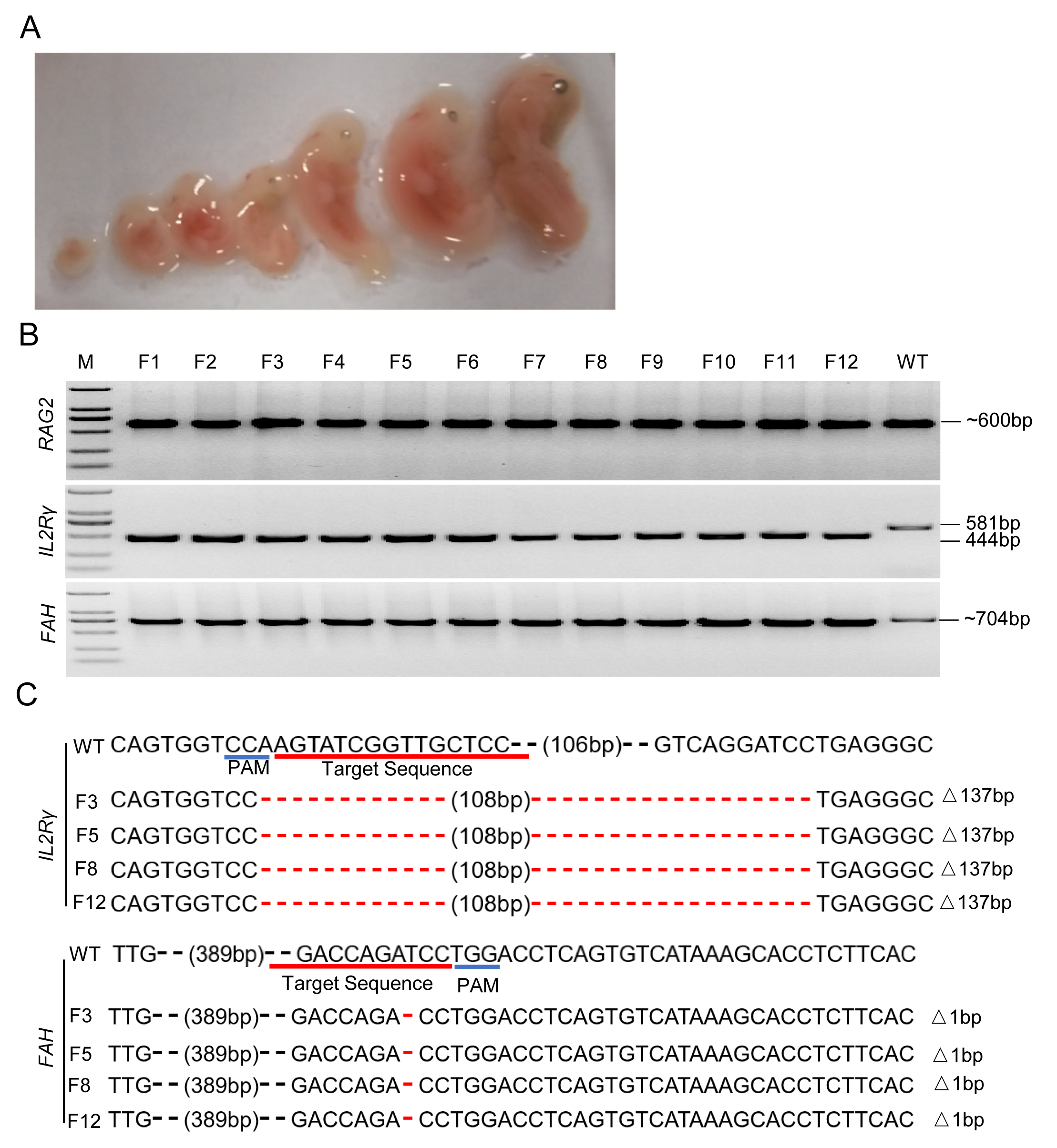

Supplement: Supplementary Figure 1 — FAH-deficiency leads to in utero fetal death without NTBC supplementation. Without NTBC supplementation during gestation, no RGFKO piglets were born. (A) Representative images of fetuses retrieved from pregnant sows implanted with RGFKO embryos at 29-35 days of gestation without NTBC supplementation. (B, C) Genomic DNA was obtained from 12 fetuses (F1-F12). RAG2, IL2Rγ and FAH were amplified by PCR (B) and amplicons of IL2Rγ and FAH were sent for Sanger sequencing. (C) Alignment of Sanger sequencing results of F3, F5, F8 and F12 with wildtype IL2Rγ and FAH sequences. 137 nucleotide deletion mutation (Δ137bp) was observed in IL2Rγ, while 1 nucleotide deletion mutation (Δ1bp) was observed in FAH. [file Image_1.tif]

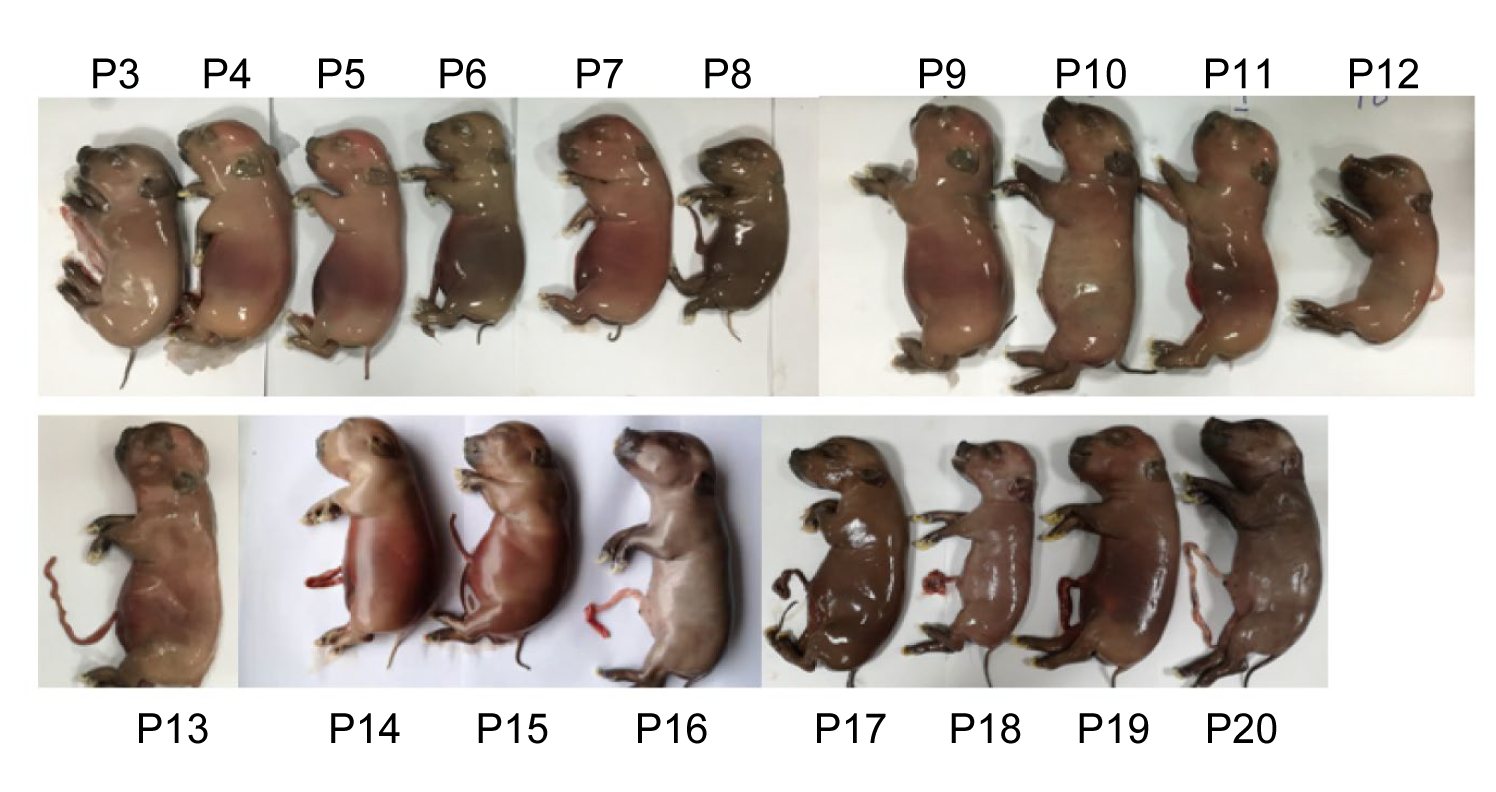

Supplement: Supplementary Figure 2 — Stillbirths of RGFKO piglets. Images of stillbirths of RGFKO piglets from three surrogate sows implanted with RGFKO embryos at 93-99 days of gestation with NTBC supplementation. [file Image_2.tif]
