# Supplementary material for: Development of RAG2 -/- IL2Rγ -/Y immune deficient FAH-knockout miniature pig
Source: Front Immunol. 2022 Aug 9;13:950194. doi: 10.3389/fimmu.2022.950194 (PMC9400017; doi:10.3389/fimmu.2022.950194)
Supplement: Supplementary Table 1 — sgRNA target sequences for RAG2, IL2Rγ and FAH. [file DataSheet_1.docx]

# Supplemental tables

| **Table S1. sgRNA target sequences for *RAG2*, *IL2Rγ* and *FAH*.** | | |
| --- | --- | --- |
| Name | Sequence (5’->3’) | Target strand |
| RAG2-sgRNA | TTGATGTCGTGTATAGTCGA | + |
| IL2RG-sgRNA | CAGCTGGAGCAACCGATACT | - |
| FAH-sgRNA | GCGATTGGTGACCAGATCC | + |

| **Table S2. Off-target sequences (OTS) predicted for RAG2, IL2Rγ and FAH.** | | | | | | |
| --- | --- | --- | --- | --- | --- | --- |
| Name | Gene | Chromosome | Position | Strand | Forward Primer（5’-3’） | Reverse Primer（5’-3’） |
| OTS-R1 | *RAG2* | Chr13 CM000824.5 | 55792653 | - | GTTAGCTTGTAGTTCCCACTTG | GTGATACAGCCAGGCAGAAG |
| OTS-R2 |  | Chr13 CM000824.5 | 161374794 | - | GTGATACAGCCAGGCAGAAG | GTGATACAGCCAGGCAGAAG |
| OTS-R3 |  | Chr13 CM000824.5 | 161601500 | - | CTGTAGTATGAAATCATCCCTG | TCACTTTGCCTTCATCCT |
| OTS-R4 |  | Chr13 CM000824.5 | 161881417 | - | TGGTAGAGTTGCCAGGAC | CCTGTAATTGTGGCTTCC |
| OTS-G1 | *IL2Rγ* | CM000815.5 Chr4 | 2450609 | + | GGATGTGCTCTTGTGGGTC | GCATCTGTAACCGACACCAC |
| OTS-G2 |  | CM000821.5 Chr10 | 10005709 | - | GCATTCACCCGCTCAGTA | CGGCTGTGGCAACTTACT |
| OTS-F1 | *FAH* | CM000812.5 Chr1 | 195936006 | + | TGTAGGTTCTTTCCTGGGTA | AGCAATGCCACTAACAGG |
| OTS-F2 |  | CM000819.5 Chr8 | 1777887 | - | TCCGAAGCCTGAGAAGACC | GCAGCAGGGAGACAGTTCA |
| OTS-F3 |  | CM000821.5 Chr10 | 41563383 | + | CCTGACCAGCCTGTTTGA | CATAATAGGAGTTCCCAGTC |
| OTS-F4 |  | CM000821.5 Chr10 | 44919247 | - | AAGCCTGGGACTCCTTAG | CTTGCGTGGGTTTAATGT |
| OTS-F5 |  | CM000825.5 Chr14 | 101886087 | + | ATCTGCTGAGCCACAATG | TCAATAGCAGCCCAACCT |
| OTS-F6 |  | CM000825.5 Chr14 | 131339903 | + | ATTGGCTTTGAAACACTCC | TGCTGTGAGCCGAGGTAT |
| OTS-F7 |  | CM000828.5 Chr17 | 30987574 | + | CTGGCATTGTAGCCTGACC | GGAAGGCAGTTACTCAAAGA |
| OTS-F8 |  | CM000830.5 ChrX | 124454124 | + | GGGCAGGAGGACAAGGAGTT | TGCAGCGAGGACCTGAATGG |
| OTS-F9 |  | AEMK02000639.1 | 183875 | + | ACCATCACTCCGGGCGACTC | CATGTGGGACCTCGGATTGA |

| **Table S3. Primer sequences used to amplify *RAG2*, *IL2Rγ* and *FAH*.** | | |
| --- | --- | --- |
| Primer name | Sequence (5’->3’) | Amplicon length |
| RAG2-PCR-F | GGGAAAACACCAAATAATGAGC | 600bp |
| RAG2-PCR-R | GTTGTCCTTGAAAGAGATGATG |  |
| ILK2RG-PCR-F | CGGCTTTTGTTTACTTGTCTGTC | 581bp |
| ILK2RG-PCR-R | ACCCACAGACGCTAAAACTAC |  |
| FAH-PCR-F | GCTGGGGTATGGTGCTAAGGATC | 704bp |
| FAH-PCR-R | AGGCGGGATGGCAGGAAGAAAG |  |

| **Table S4. Primer sequences used for qPCR.** | | |
| --- | --- | --- |
| Primer name | Forward Primer (5’->3’) | Reverse Primer (5’->3’) |
| IL2RG-qPCR | ATTGTCGGCCTCATGTGTGT | ACTCCAGGCCGAAAAGTTCC |
| CD4-qPCR | GGCAAAACCACAATGGACCC | GCGAGATCTCCTGCCTTACC |
| CD8-qPCR | CGTCTTCTTGCCAGCAAAGC | GCGCCCAGTTGTACAGATCA |
| CD19-qPCR | CAGGATGGCAGCAACTACGA | GGCTCAGGAAGTCTGTCGTC |
| GAPDH-qPCR | TCGTGGAGGGACTCATGGTAGG | TACCCCTCCTCTGATGTCCTGAG |

| **Table S5. Primer sequences used for V(D)J rearrangement detection.** | | |
| --- | --- | --- |
| Primer name | Forward Primer (5’->3’) | Reverse Primer (5’->3’) |
| IgH-V-PCR | GCCATGCAAATGGGTTCCTC | AGGCTTCACCTCTGTCGATG |
| IgH-VDJ-PCR | GCCATGCAAATGGGTTCCTC | CCCAGAGATCCATAGCATAG |
| TRB-V-PCR | GATGTCATGGACATCATTTGCCATC | ATAGGCCATCATTGACTTCC |
| TRB-VDJ-PCR | GATGTCATGGACATCATTTGCCATC | CTCCAAAGAAGACTTCAGTG |
| TRD-V-PCR | TTCAGACACGTGACCTTCAG | GCTGAGATTCTGGGATTCAC |
| TRD-VDJ-PCR | TTCAGACACGTGACCTTCAG | GTTCCACAACCAGCTGAGTC |

**Table S6. Biochemical function of liver.**

| Group | ALT | AST | ALP | TBIL | DBIL | TP | ALB |
| --- | --- | --- | --- | --- | --- | --- | --- |
| RGFKO | 81 | 4785 | 490 | 26.14 | 10.07 | 51.6 | 30.3 |
| WT1 | 25 | 44 | 529 | 2.00 | 1.30 | 63.7 | 35.3 |
| WT2 | 20 | 62 | 914 | 0.70 | 0.50 | 61.7 | 37.5 |
